# Development of a Three-Dimensional Pathology-Simulating Model of Neurotrauma Using a Polymer-Encapsulated Neural Cell Network

**DOI:** 10.3390/gels11040247

**Published:** 2025-03-27

**Authors:** Jessica Patricia Wiseman, Zoe Dombros-Ryan, Jack Griffiths, Christopher Adams, Divya Maitreyi Chari

**Affiliations:** 1 Faculty of Biology, Medicine and Health, University of Manchester, Manchester M13 9PL, UK; jessica.wiseman@manchester.ac.uk; 2 School of Medicine, Keele University, Staffordshire ST5 5BG, UK; z.dombros.ryan@keele.ac.uk; 3 School of Life Sciences, Keele University, Newcastle ST5 5BG, UK; x8i47@students.keele.ac.uk

**Keywords:** traumatic brain injury, in vitro models, 3D modelling, brain pathology, scarring, immune responses, therapeutics, hydrogels

## Abstract

Penetrating traumatic injuries of the brain have a poor clinical prognosis necessitating development of new therapies to improve neurological outcomes. Laboratory research is hampered by reliance on highly invasive experimental approaches in living animals to simulate penetrating injuries e.g., by cutting/crushing the brain tissue, with a range of associated ethical, technical and logistical challenges. Accordingly, there is a critical need to develop neuromimetic in vitro alternative neural models to reduce harm to animals. However, most in vitro, reductionist simulations of brain injury are too simplistic to simulate the complex environment of the injured nervous system. We recently reported a complex, two-dimensional in vitro mouse model of neurotrauma containing five major brain cell types to replicate neural architecture, grown on a “hard” glass substrate in a brain cell sheet. We now demonstrate the translation of this approach into a three-dimensional tissue injury model, by propagating the entire cellular network in a “soft” compliant collagen hydrogel, similar to native brain tissue stiffness (an important determinant of cell fate). A multicellular network of neural cells was observed to form in the polymer matrix containing all major brain cell populations, including the immune cells (microglia). We demonstrate that it is feasible to create a reproducible, focal traumatic injury in the synthesised neural tissue construct. Importantly, key pathological features of neurological injury, such as astrocyte scarring, immune cell (microglial) activation, impeded axonal outgrowth and stem/progenitor cell migration, can be successfully induced. We also prove that it is feasible to implant a biomaterial into the lesion gap to study neural cell responses for screening applications. The findings support the concept that the model can be used in a versatile manner for advanced neural modelling.

## 1. Introduction

Live animal models of penetrating neurotrauma involve some of the most invasive procedures in experimental biology, with profound neurological deficits induced (hemiplegia, quadriplegia, bladder dysfunction and infection risk) [1,2]. The models require extensive training, infrastructure, regulatory licenses and facilities for pre/post-operative care. Induction of injuries can be variable across groups, and do not allow for reliable statistical comparisons across groups without large animal numbers. For developmental screening applications, such invasive models present significant ethical dilemmas, with public opinion warranting that alternative approaches be developed to reduce reliance on animal testing [3]. Pathology-mimetic and higher-throughput alternatives are urgently needed for biological testing, in line with the global drive for Reduction, Refinement and Replacement (3R’s) of animal research.

Despite this high need, in vitro alternatives to live animal neurological models are generally overly simplistic, lacking the multicellular brain cell network found in vivo, and usually lack an immune (microglial) component, or complex neuronal network, although protocols are under development to add in microglia using additional culturing steps. Accordingly, they are unable to mimic cardinal pathological features of neurological injury or be used to study post-injury regeneration. In vitro models range in complexity from the simplest, two-dimensional (2D) monolayer cell cultures to the highly complex three-dimensional (3D) organotypic slice or organoid cultures. Different models have specific associated features which determine their research utility in biomaterials research, e.g., pathological relevance, technical difficulty and ease of maintenance [4]. Organotypic slice (OTS) cultures represent a transitional system that preserves the original tissue, and its inherent structural architecture, combining benefits of in vivo and in vitro cell culture models. An advantage of this approach is that it permits therapeutic agents/biomaterials, stimuli or mechanical/chemical injury to be applied directly to the slice at any stage of cultivation. These offer a moderate-throughput platform to simultaneously monitor parameters of neural regeneration (i.e., nerve fibre outgrowth, glial scar formation, remyelination and immune cell activation) in response to various injury mechanisms and therapeutic approaches [5].

Alternatively, there has been a large focus on human iPSCs to generate neural cells in 2D in vitro culture, including in combination with microfluidic culturing systems to generate brain-on-a-chip modelling systems resembling tissue-like physiology [6]. These systems are generally low-throughput and time-consuming with limitations to their scalability and lack cellular maturity within the models. Human cell-derived CNS organoids are another complex in vitro modelling contender, which have potential for molecular/structural mimicry of CNS tissues. CNS organoids are stem cell-derived self-organising suspension cultures with major neural cell types (generally excluding microglia) and cytoarchitectures recapitulating developing tissues [7]. Self-organising approaches allow for the formation of a “mini-brain” displaying multiple regions of neural tissue comparable to the human brain [8] with integration into organoid “assembloids” through fusion of separate organoid types to generate complex systems. In terms of recapitulating traumatic injury, organoid techniques do not provide an accessible platform for physical manipulation due to their free-floating nature. However, one group in 2020 developed a mechanism of traumatic injury to brain organoids through high-intensity focused ultrasound [9].

Hydrogel-based polymeric biomaterials have emerged as a major platform to mimic the extracellular matrix of soft tissues for the development of 3D CNS-like tissue models, as their high porosity allows free movement of fluid and nutrients for 3D cell growth. Hydrogels can be modulated to match the endogenous tissue stiffness of neural tissues (which can vary by anatomical region (3–10 kPa)) [10] following injury. Cell-encapsulating hydrogels have the potential to be adapted for development of high-throughput modelling systems with technical ease whilst offering complex in vivo-like cellular dynamics. Despite this, few studies have used hydrogels for growth of complex neural cell networks within which to simulate neural trauma.

Here, we have deployed a collagen-based hydrogel matrix to demonstrate that:

(a) growth of a complex multicellular neural network can be achieved within the polymer substrate;
(b) a focal area of trauma can be reliably and reproducibly induced in the construct;
(c) complex injury pathology can be simulated; and
(d) the model can support therapeutic testing, by implanting a surgical grade biomaterial into the lesion focus.

Based on our findings, we propose that this new approach can be used as a high-throughput 3D injury model to evaluate neural cell responses to introduced therapeutics.

## 2. Results

### 2.1. A 3D Neural Cell Network Was Observed to Develop in Hydrogels over Time

**Figure 1:**
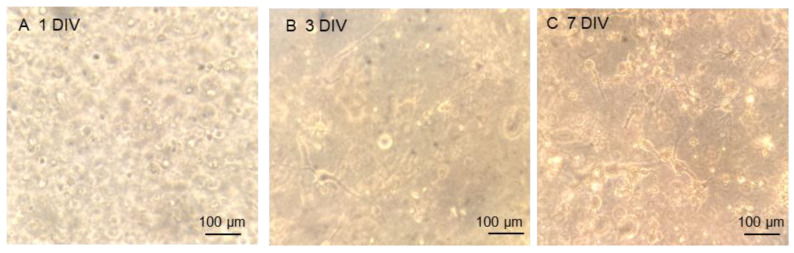
Brightfield images of cell network development over 7 days in culture: representative micrograph at (**A**) 1 DIV where cells remained rounded up; (**B**) 3 DIV where cellular processes begin to emerge; and (**C**) 7 DIV where a network of cells is emerging with distinct cellular morphologies observed.

Phase contrast microscopy was used to examine the formation of a cellular network within the gel constructs. Cells remained rounded at first (Figure 1A), but by 3 days in vitro (DIV) some cellular processes could be identified projecting from cellular bodies (Figure 1B). Some cellular morphologies could be identified here through the fields of focus. By 7 DIV multiple cellular morphologies could be identified across the gels (Figure 1C).

### 2.2. All Major Neural Cell Populations Could Be Detected in the 3D Neural Constructs

**Figure 2:**
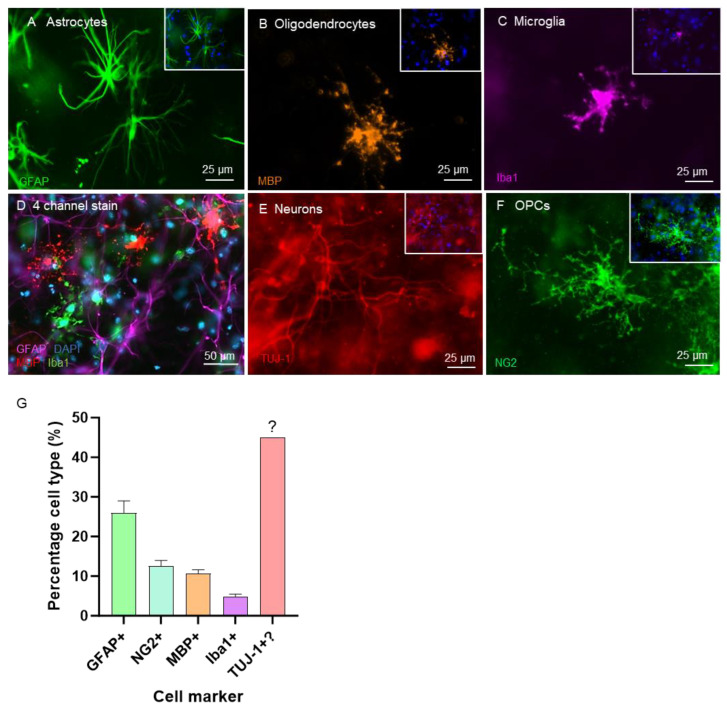
Cellular morphology of the five major neural cell types encapsulated within the 3D hydrogel construct: representative fluorescent micrographs demonstrate (**A**) fibrous (GFAP+) astrocyte morphologies, (**B**) mature MBP+ oligodendrocyte morphologies, (**C**) ramified “resting” Iba1+ microglia, (**D**) quadruple-stained micrograph revealing a poly-glial network of astrocytes, oligodendrocytes and microglia, (**E**) presence of a TUJ-1+ neuronal network and (**F**) highly processed NG2+ OPCs. Bar graph in (**G**) demonstrates the cellular distribution in the construction; neuronal populations were not quantifiable due to the dense network of nerve fibres rendering identification of individual neurons challenging, and therefore estimated by a subtraction process in the graph [*n* = 4].

To examine the neural cell types in further detail and evaluate how their morphology was influenced by the 3D hydrogel, high-magnification immuno-stained images were analysed. A complex network of five major brain cell populations could be detected within the 3D construct. GFAP+ astrocytes displayed many fine long processes from a central body (Figure 2A). MBP+ oligodendrocytes presented multiple-processed and branching morphologies (Figure 2B). Iba1+ microglia presented ramified morphologies indicative of a resting phenotype (Figure 2C). Additionally, 4-channel staining confirmed the presence of a complex poly-glial network in the gel (2D). Though harder to visualise than the other cell types, a TUJ-1+ neuronal network could be detected against a dense background (Figure 2E). NG2+ OPCs exhibited large and highly processed morphologies with a large surface area (Figure 2F). The proportions of each cell type were quantified and are shown in Figure 2G. Neuronal distribution was estimated within the 3D model via a subtraction process; the ~45% of nuclei that were unaccounted for were presumed to be neurons.

### 2.3. Cell Morphology and Distribution Patterns of Astrocytes in the Gel Constructs Showed Distinct Phenotypes

**Figure 3:**
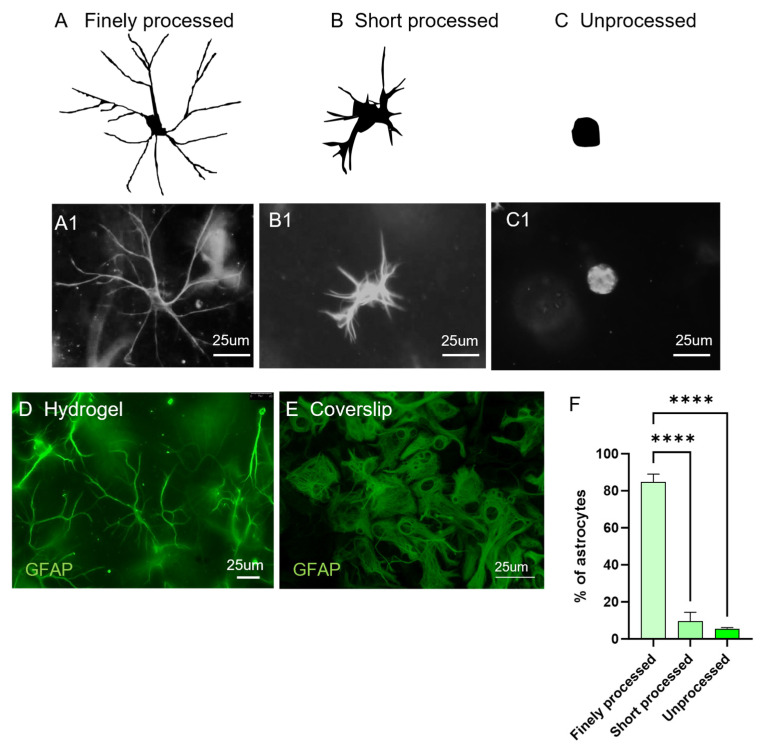
Astrocyte morphology analysis in 3D culture: three distinct astrocytic morphologies were identified: (**A**) finely processed, (**B**) short-processed and (**C**) unprocessed. Representative diagrams are individual traces from micrographs in (**A1**–**C1**). Representative fluorescent micrographs of GFAP+ astrocytes throughout the gel (**D**) compared with the standard pattern of cell growth on coverslips (laboratory data unrelated to this study) (**E**). Bar graph (**F**) displays the distribution of astrocytic morphologies [one-way ANOVA, with Bonferroni’s multiple comparison test (MCT), **** *p* < 0.0001, *n* = 4].

To investigate the impact of intra-gel growth on astrocytic morphology, a detailed morphological analysis was performed on GFAP+ cells. Three distinct morphologies were identified; the most abundant were long fine processes emerging from a central cell body (Figure 3A,A1). Less frequently observed were astrocytes with very short, restricted processes (Figure 3B,B1) or unprocessed spherical astrocytes (Figure 3C,C1). Finely processed astrocytes were evenly distributed throughout the gels and made up the majority (>ca 80%) of the observed astrocytes (Figure 3D). Additionally, the morphology of the gel encapsulated GFAP+ cells differed from those grown on hard coverslips. The 2D morphologies showed classic flattened, polygonal morphologies and lacked the fine processes that were observed in 3D cultures (Figure 3E). Quantification of the individual morphologies is shown in Figure 3F, with the branching phenotypes showing a significantly higher proportion. It was found that 84.8 ± 2.4% of astrocytes were finely processed, 9.5 ± 2.7% of astrocytes were short-processed and 5.5 ± 0.3% were unprocessed, with the difference found to be highly significant (Figure 3F).

### 2.4. Generation of Focal Injury Within the 3D Cellular Construct

**Figure 4:**
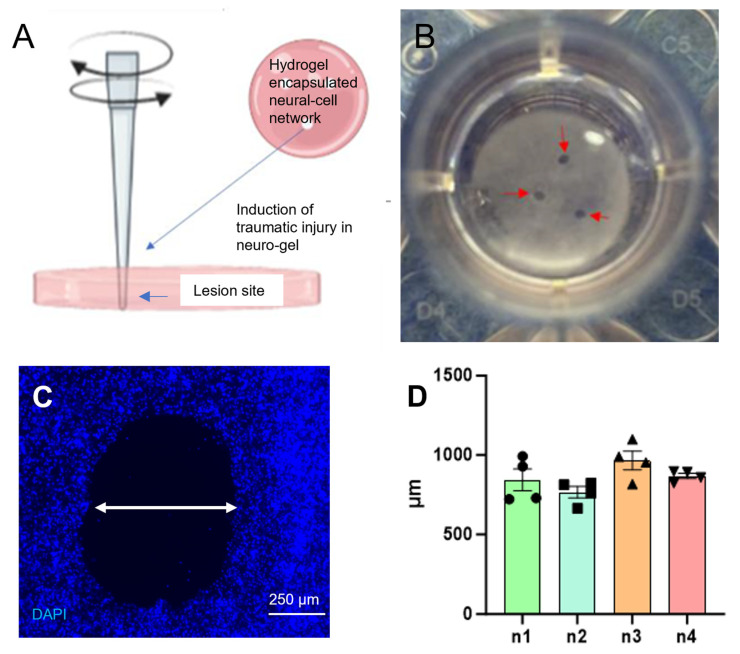
Illustration of the introduction of a focal injury in the gel construct: (**A**) shows the polymer construct, a schematic representation of focal injury method and the lesion site examined in histological assays; (**B**) shows injured gel in culture medium showing multiple, focal lesions with reproducible injury diameters under light microscopy and (**C**) shows representative fluorescent micrograph at the injury site with DAPI staining of nuclei (blue) and lesion width indicated (arrow). (**D**) graph showing the reproducible injury diameter per biological repeat [*n* = 4].

The quality and consistency of the focal penetrating injuries was assessed by measuring the diameter of lesion sites. The injury induction process is shown schematically in Figure 4A. It was feasible to create multiple, distinct injury cavities that were easily detectable, supporting the analysis of injury responses (Figure 4B,C). At 1 day post lesion (DPL), the average injury diameter per gel across multiple repeats was 860.12 ± 41.01 µm (Figure 4D).

### 2.5. A Focal Injury Within the 3D Neuro-Construct Induced Injury-Specific Responses

**Figure 5:**
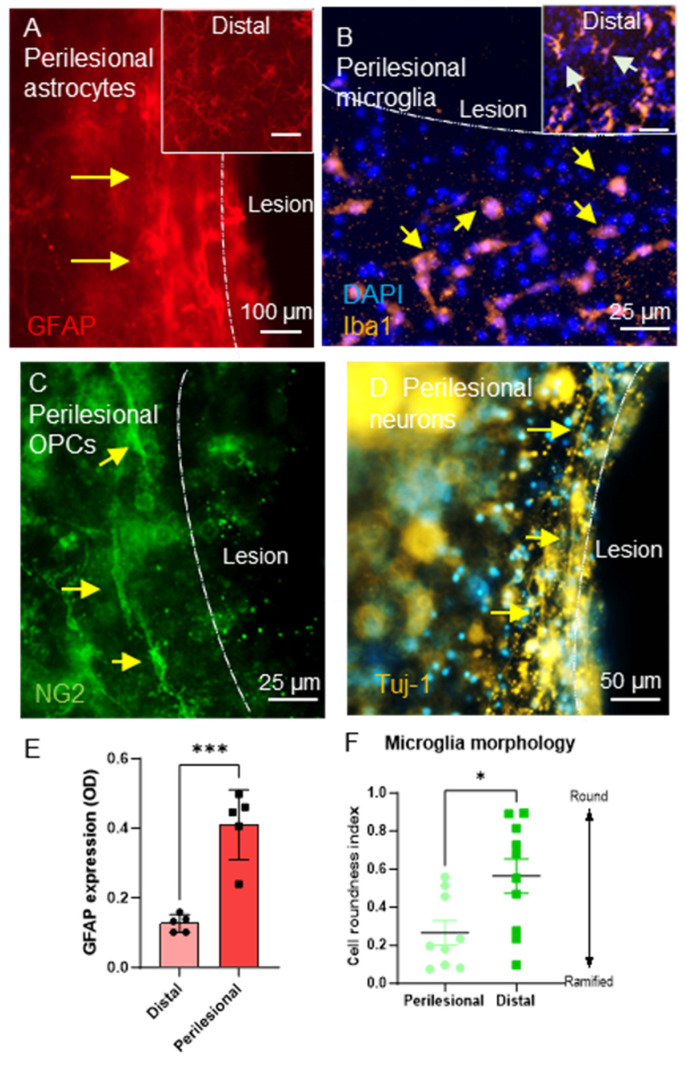
Key pathological responses could be detected in response to the focal injury: (**A**) Injury-induced astrogliosis proximal to the lesion cavity: representative fluorescent micrograph representing GFAP+ astrocytes at the lesion margins upregulating GFAP expression (arrows). Inset shows branching morphologies of distal astrocytes. (**B**) Injury-induced microglial reactivity observed adjacent to the lesion cavity within gels. Mostly amoeboid/bipolar morphologies observed near the injury cavity (yellow arrows) compared with ramified phenotypes of distal cells (inset, arrows). (**C**) OPC morphologies adjacent to the lesion. Distinct bipolar migratory morphologies shown (arrows). (**D**) Representative micrograph from a pilot study showing perilesional neuronal growth around the lesion, arrows [*n* = 1]. (**E**) Bar graph reveals a significant increase in GFAP expression of perilesional astrocytes (unpaired *t*-test, *** *p* < 0.001, *n* = 4). (**F**) Graph showing perilesional vs. distal iba1+ cell assessment on the cell roundness index [Unpaired *t*-test, * *p* < 0.05, *n* = 3, each point on the graph represents one microscopic field].

The pathomimicry of the injury model was assessed by examining the pathological response of the cells at the lesion site. GFAP+ astrocytes displayed injury-induced astrogliosis at the lesion margins, evidenced by a visually substantial increase in GFAP expression (Figure 5A, arrows show the band of intense GFAP staining) versus distal cells (Figure 5A, inset). An injury-activated microglial response was also observed; microglia proximal to the injury site displayed rounded (amoeboid) morphologies post-injury (yellow arrows, Figure 5B) versus their distal counterparts (Figure 5B, inset, arrows show ramified phenotypes). NG2+ OPCs adjacent to the lesion displayed bipolar migratory morphologies suggestive of a migratory phenotype (Figure 5C, arrows). In a pilot study (*n* = 1), we observed some evidence of a neuronal response to the injury with neuronal projections tending to grow around the lesion rather than toward the injury focus (Figure 5D, arrows). Further investigation was made impossible due to the amount of debris and non-specific staining in the remaining samples.

Optical density measurements of perilesional astrocytes were assessed to determine the degree of injury-induced astrogliosis. The upregulation of GFAP at the injury margins showed a significant increase (ca 3-fold) (0.41 ± 0.045 OD) versus the baseline level GFAP expression of distal astrocytes (0.127 ± 0.011 OD) (Figure 5E). The microglia cell roundedness index (CRI) revealed that perilesional microglia (0.266 ± 0.064 CRI) were significantly more rounded than their distal counterparts (0.564 ± 0.091 CRI) at 3 DPL (Figure 5F).

### 2.6. DuraGen^TM^ Implantation in the Injury Cavity Demonstrated Microglial Infiltration

**Figure 6:**
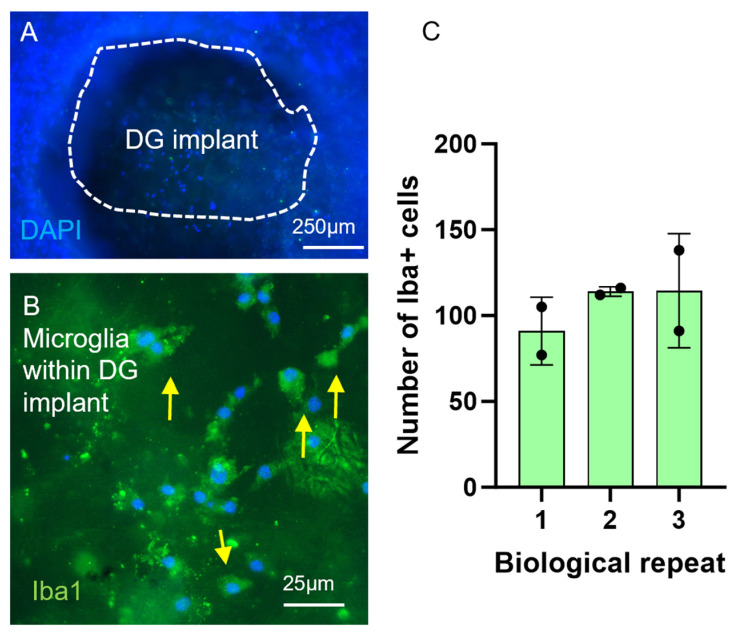
DuraGen^TM^ (DG) biomaterial implantation induces a microglial response. Nuclei (DAPI) within the implanted DG (white dashed lines) within the injury cavity at 3 days post-injury (**A**) and Iba1+ microglia cells (arrows) within the implant (**B**). (**C**) Graph showing quantification of Iba1+ cell infiltration per unit area of implanted DG; circles indicate number of implants in each biological repeat.

The injury process reproducibly induced a defined cavity in which to implant a biomaterial immediately after injury. At day 3 post-injury, multiple nuclei were detected within the DuraGen^TM^ implant (Figure 6A). These were demonstrated to be activated microglia migrating in response to injury and biomaterial implantation. The infiltrating cells stained positive for Iba-1 and amoeboid morphologies could be detected in the implanted material suggesting an activated state (Figure 6B). The number of Iba1+ microglia within the DuraGen^TM^ was estimated to be 106.5 ± 7.8 microglia cells per unit area of the implant (Figure 6C).

## 3. Discussion

The data presented here demonstrate that it is feasible to develop a dense network of five major brain cell populations within a soft polymer matrix, in order to develop a neuromimetic brain tissue plug. We also show that a localised, highly reproducible traumatic injury can be created in this neuro-construct. The results demonstrate a functioning 3D injury model within which stereotypical injury responses (seen in vivo following traumatic injury) can be induced. To our knowledge, this is the first time these key cellular pathological responses have been reported within a 3D neural construct model. Key observations post-injury are as follows: induction of a glial cell scar (a known barrier to axonal regeneration) with elevated GFAP expression; induction of amoeboid microglial phenotypes indicative of activation and bipolar, potentially migratory OPC profiles at the lesion margins (as reported in vivo following traumatic brain injury) [11]. Imaging of the axonal network around the injury site proved to be challenging; however, in proof-of-concept studies, axons tended to grow around the injury rather than towards it, suggesting a barrier to regeneration; we can speculate due to the glial scar. Critically, we were able to show that a mouldable biomaterial (surgical-grade dural sealant Duragen Plus^TM^) could be introduced into the lesion cavity, to simulate biomaterial implantation in vivo, with extensive cellular infiltration by microglia.

In terms of traumatic brain injury models, Liaudanskaya, et al. (2020) [12] reported a controlled impact study on a brain-like tissue model of a 3D cortical neuronal culture through an established impact device. Other studies have involved calcium-dependent injury and oxygen/glucose-deprivation injury on 3D constructs of primary human NSCs [13] or the use of a compression device on cortical neuronal 3D hydrogels [14]. We are not aware of similar studies investigating penetrating traumatic brain injury in the 3D context, or induction of pathology and biomaterial interfacing with neural cell populations.

We believe the model offers a range of advantages versus current available in vitro neural model systems. Please note that while technologies such as human iPSCs and brain organoids have revolutionised neural cell models, we consider that our model offers several comparative advantages in terms of scalability, presence of immune cells, cellular maturity, accessibility and ease of injury induction/imaging, while also avoiding issues associated with core necrosis in dense 3D models [15]. First, the dissociated tissue to develop the model is derived from primary cells which limits drawbacks associated with the widespread use of neural cancer cell lines (such as abnormal chromosomal number, cryptic contamination, aneuploidy and resistance to toxicity) [16]. The cells are derived from postnatal mouse tissue, avoiding the sacrifice of breeding females to derive embryonic tissue. It offers a high degree of cellular complexity in order to mimic in vivo neural architecture and the interactions of multiple brain cell populations, including the native immune cells. Additionally, all cell types are derived simultaneously, from the same tissue, and are treated identically throughout the process, limiting variability associated with deriving cells from different sources. For example, in 2020 Raimondi et al. [17] reported a 3D brain-like tissue model from primary cortical cells, wherein a co-culture of independently isolated glial cells and neuronal cells was carried out. This involves a time-consuming process to derive each cell type individually (e.g., 20 days to harvest astrocytes from a confluent flask). By contrast, we can achieve a complex neural network in approximately 7–20 days.

Second, a key feature of our model is in vitro simulation of cardinal neuropathological events in vivo, highlighting the utility of the model as a core approach to screen therapeutic interventions prior to in vivo testing. Focal injuries could be induced in a highly reproducible manner in terms of size/shape. Additionally, multiple injuries can be induced in the same construct, so several experimental replicates can be generated in parallel for multiple comparisons of control/test conditions. The model can also be adapted into a high-throughput format adaptable to different well formats and construct sizes. The 3D constructs are amenable to imaging using standard microscopy methods such as Z-stack imaging and confocal microscopy, and dynamic live cell imaging with quantification of cells and pathological responses feasible.

Third, we consider that the approach is highly versatile and can potentially be adopted in the future for a range of pathologies (e.g., contusion injuries or demyelination), anatomical areas (example cortex versus cerebellum), species (rodent including genetically modified species, induced pluripotent stem cells, large animals and human tissue), tissue ages (to simulate neonatal versus adult tissue and pathology), different polymers for encapsulation (to study the effects of tailored biomaterials on neural cell development) and biomaterial implantation (for screening of a range of nanomaterials and biomaterials). Polymer concentrations can be adjusted so as to mimic human brain tissue stiffness, to create a more physiologically relevant system versus free floating models or cells grown on “hard” substrates (e.g., glass). For example, we previously reported that collagen gels with a similar concentration to this study (0.6–1.2%) had stiffnesses ranging from 34 to 150 Pa [18], which is similar to the stiffness of embryonic brain. This is important as it is increasingly recognised that substrate stiffness can critically impact cell fate and development, so stiffness matching to host tissue is critical; as an example, neurons show greater elongation on soft substrates [19]. Additionally, elegant studies have demonstrated that stiffness of injured neural tissue differs from that of normal tissue [20]. Our model offers the capacity to tune the polymer stiffness to mimic such variables. It is noteworthy that the cellular proportions established within the soft, 3D constructs differed from those in a 3D network on a hard substrate (3D: astrocytes: 25.97 ± 3.1%, neurons: 45% estimated, oligodendrocytes: 10.7 ± 0.85%, microglia: 4.77 ± 0.69% and OPCs: 12.5 ± 1.48% versus 2D: astrocytes: 35.3 ± 0.2%, neurons: 45.8 ± 2.1%, oligodendrocytes: 2.73 ± 0.17%, microglia: 10.35 ± 3.89%, OPCs: 11.15 ± 0.32%), suggesting an influence on cell fate [21].

Finally, the model is technically easy to develop and monitor, scalable and very cost effective compared to live animal experiments. In our experience, users can be rapidly trained (2–3 weeks), making it accessible to users across the world, including low–middle-income countries, without requiring special regulatory permissions e.g., from the Home Office. Accordingly, we believe that this approach can offer a valuable new tool for neurobiology research and the development of neurotherapeutics.

In the future, it would be valuable to establish if clinical imaging techniques, such as magnetic resonance imaging or magnetic resonance spectroscopy, can be used to monitor and study pathology in situ. The development of 3D multielectrode arrays (for example, containing pillars with embedded electrodes [22] for electrophysiological readouts from the tissue plug) would also provide valuable functional readouts for real-time multimodal monitoring of the constructs, in parallel with the ability to monitor cellular pathology using histological assays.

While NG2+ OPCs maintained their multi-processed morphologies and ramified Iba1+ microglia were expressed in distinct patches (albeit at lower numbers than the 2D model), a striking observation was the alteration in astrocytic phenotypes compared with cells on 2D glass substrates. It is not clear what accounts for this observation. Astrocytes are known to remodel their environment [23], and it is feasible that the process is associated with dramatic alterations in their morphologies. It will be of high value to carry out detailed genomic/proteomic profiling of the polymer-encapsulated cells to (a) understand and quantify the difference in gene/protein expressions which could contribute to altered cell fate and (b) to compare these with in vivo gene expression (to study if 3D models in soft substrates can more closely mimic in vivo neural microenvironments) versus standard neural cell culture on hard substrates. Whilst it was out of the scope of this study, future experiments may also attempt to study the complex cell–cell interactions which are known to influence ongoing pathological response in neural injury [24]. Our system may offer a simpler format to achieve this than in vivo models where spatiotemporal resolution of cell behaviour is much harder to achieve.

## 4. Conclusions

We have successfully scaled our 2D model [25] by demonstrating fabrication of a 3D neural model containing all the cell types of the cortex grown within a soft polymer matrix to mimic the mechanical properties of the brain. Further, we have characterised neural cell morphology within the model and, importantly, cardinal pathological features can be recapitulated in the model in response to a traumatic injury. Proof-of-principle experiments also indicated novel neurotherapeutics could be delivered into the injury site to test their efficacy. Our 3D model could represent a powerful tool to study neural cell responses to/handling of therapeutics (e.g., drugs or biomaterials) delivered directly to neural tissue. In terms of limitations, the model does not currently have an embedded vasculature, or the ability to simulate the blood–brain barrier, which imposes limitations on delivery of neurotherapeutics. However, advances in the development of biomimetic hydrogel-based blood vessel models [26] could be used to engineer a functional vasculature through the constructs, potentially in combination with flow systems, enhancing the screening and neural modelling utility of the approach.

## 5. Materials and Methods

### 5.1. Materials

Culture plastics and media were from Thermo Fisher Scientific (Loughborough, UK) or Scientific Laboratory Supplies (SLS, Nottingham, UK), unless stated otherwise. Vectashield mounting medium containing 4′,6-diamidino-2-phenylindole (DAPI) was from Vector Laboratories, UK. Duragen Plus™ matrix (medically approved, neurosurgical-grade biomaterial derived from Type I bovine collagen) was from Integra LifeSciences, Princeton, NJ, USA.

### 5.2. Antibodies

Primary antibodies were rabbit anti-glial fibrillary acidic protein (GFAP), from DakoCytomation, (Ely, UK), goat anti-ionised calcium-binding adapter molecule 1 (Iba1) from Thermo Fisher Scientific (Loughborough, UK), mouse anti-nerve/glial antigen 2 (NG2) and rat anti-Myelin Basic Protein (MBP) from Millipore (Danvers, MA, USA), mouse anti-beta-III-tubulin (Tuj1) from Biolegend (San Diego, CA, USA). Secondary antibodies were fluorescein isothiocyanate (FITC)-conjugated donkey anti-mouse, -rabbit, -goat; cyanine 3 (Cy3)-conjugated donkey anti-mouse, -goat, -rat; cyanine 5 (Cy5)-conjugated donkey anti-mouse and -rabbit from Stratech Scientific (Suffolk, UK).

### 5.3. Preparation of Mixed Cortical Brain Cell Cultures

Fresh tissues were dissected from mouse pups (CD1), with litters ranging from 8 to 12 pups. Keele University retains Home Office licensed authority, as a designated premises, providing regulatory compliance for the care and welfare of the animals used in this study [Keele University Establishment licence number: X350251A8 (copy available on request)]. Ethical approval for the schedule 1 usage of animals used in this study was obtained from Keele University Animal Welfare and Ethical Review Body in 2017. Mice maintaining specified pathogen-free health status were housed and bred in the Keele Biological Service Unit, in accordance with the Code of Practice for the Housing and Care of Animals Bred, Supplied or Used for Scientific Purposes. Litters were maintained on a continuous 12:12 light cycle, 22.5 ± 0.4 °C, 46% ± 5% humidity. Mice were bred and maintained according to the UK Code of Practice for the housing and care of animals used for scientific procedures, Animals (Scientific Procedures) Act 1986. Pups of both sexes were used in the study and culled via the schedule 1 method of an overdose of anaesthetic, sodium pentobarbital (Animalcare Ltd., York, UK), 1 mL/kg intraperitoneal injection, on post-natal day 1–4, weight ca 2.5–3.5 g.

Brains were dissected and transferred to dissection medium (2.5% HEPES in Earl’s balanced salt solution) on ice. Under laminar flow, cerebral cortices were isolated by removing the olfactory bulbs, hindbrain, and subcortical tissue. Cortical rolling on sterile paper removed the meninges and blood vessels. Cortices were minced with a scalpel and pelleted at 1200 rpm for 5 min, then resuspended in dissection medium to cover the pellet. Then, 0.25 mL of DNase and 0.5 mL Trypsin-EDTA was added to every 4 brains dissected. The cells were then shaken at 37 °C for 20 min at 150 rpm. Cells were gently triturated to further break any tissue aggregates by a P1000 (without frothing), and 2 mL of fetal bovine serum added to stop cell-trypsinisation. 2 mL of neurobasal medium (96% Neurobasal A (48 mL), 2 mM Glutamax-I (0.5 mL), 2% B27 (1 mL), 50 μg mL^−1^ penicillin, and 50 μg mL^−1^ streptomycin (0.5 mL) was used to triturate the cells (×30), which were then pelleted at 1200 rpm for 3 min, resuspended in 2 mL of neurobasal medium and filtered into 50 mL tubes using 70 μm then 40 μm cell strainers, with rinsing using neurobasal medium. The resulting cell stock was diluted in trypan blue (1:5), which can pass through the membrane of dead cells, dyeing the cytoplasm blue, while it does not stain live cells. The solution was added to a hemocytometer and a manual count of the live and dead cells was completed on a microscope. All further calculations were performed based on the number of viable cells observed.

### 5.4. Construction of 3D Cellular Hydrogels

Gel formulation was adapted from Adams et al., 2016 [18]. For the cellular 3D gels, the enzymatically dissociated cortical dissociate was incorporated into the collagen solution prior to setting, allowing cortical cells to be encapsulated within the collagen fibres as part of a 3D network. Gels were set in 24 well plates on top of glass coverslips treated with EtOH (5 min), washed with water and left to air dry. The coverslips were found to facilitate the removal of gels from the wells. Final seeding densities of 2.5 × 10^7^ cells/mL with collagen concentrations of 1 mg/mL were used to establish the gels. To generate certain collagen concentrations within the gel, a set of formulae were used to calculate volumes of each reagent [18]. Firstly, the collagen was dissolved in acetic acid to the required concentration, then 10 × MEMα solution was added. Subsequently, the appropriate volume of cells was added, which needs to be immediately titrated throughout the solution or it can cause aggregations. Lastly, the solution was neutralised with NaOH, and this was added drop wise whilst at the same time swirling the mixture until the pH indicator (within the MEMα) turns from yellow to pink. All components were always kept on ice to ensure the gel did not set before seeding into wells. Then, 200 µL of the gel solution was added to sterile glass coverslips, which forms a button shape. Once seeded, plates were transferred carefully to the incubator for 30–60 min. Following gelation, 500 μL of complete neurobasal medium was added to each well. The gel medium was topped up to 1 mL after 2 days and had a 50% medium change every 3–4 days.

### 5.5. Introducing an Injury into the 3D Cellular Gels

Once the gels had been in culture for a minimum of 7 days, a network of cells could be visualised under light microscopy. The injury was made when gels appeared relatively confluent with cellular processes extending in most fields of focus. The tip end of a P1000 pipette tip was used to puncture through the gel then twisted clockwise and anticlockwise several times until the gel was completely cut. The waste gel was held inside the pipette tip end and removed. This was carried out 3 times to create 3 separate lesions within the same 3D cellular gel culture.

### 5.6. Implantation of a Biomaterial Within the Injury Site of the Gels

DuraGen^TM^ (Integra LifeSciences, Princeton, NJ, USA) was cut to 900 µm wide strips by the tissue chopper, then each strip was cut to size with a scalpel to fit the injury within the gel. The medium was removed, and the soaked material was implanted into the injury area using forceps; the wells were then refilled with 2 mL of medium (the material expands to fill the injury once medium added).

### 5.7. Fixing and Staining the Gels

At appropriate timepoints, gels were fixed with 4% PFA for 30 min. Post-fixation, the PFA was removed, and the gels were washed 3 times with PBS (10 min per wash). The gels were blocked for an hour with 5% normal donkey serum with 0.3% triton X-100 (Merck, Gillingham, UK) in PBS. See Section 5.2 for details of antibody suppliers. Primary antibodies chosen to stain for the 5 major cell types encapsulated in the gels were the following: mouse anti-Tuj1 (1:1000 in blocking solution) for neurons, rabbit anti-GFAP (1:500) for astrocytes, rabbit anti-Iba1 (1:200) for microglia, rabbit anti-Ng2 (1:200) for OPCs and rat anti-MBP (1:200) for oligodendrocytes. The primary antibody solutions were applied for 2 days at 4 °C. The gels then underwent 3 × 1 h washing steps in PBS. Following this, gels were incubated with the secondary antibodies which corresponded to the primary antibodies selected (FITC-conjugated donkey anti-mouse, -rabbit, -goat; Cy3-conjugated donkey anti-mouse, -goat, -rat and Cy5) conjugated donkey anti-mouse and –rabbit; 1:200 in blocking solution) with 2 µL/mL of DAPI for 6 h at RT and then washed 3 times at 1 h per wash with PBS. Subsequently, gels were mounted on glass slides with mounting medium (no DAPI).

### 5.8. 3D Culture Analyses

Fluorescence imaging and z-stack imaging of the gels was carried out on the Zeiss, Axioscope A1 microscope with an AxioCam ICc1 digital camera processed with Axiovision software (v3.2) (Carl Zeiss Microimaging GmbH, Goettingen, Germany). Quantitative analysis was performed using an Axio Observer.Z1 equipped with an AxioCam MRm powered by Zen 2 (blue edition) software (Carl Zeiss MicroImaging GmbH, Goettingen, Germany). All pathological responses to induced injuries were documented at 3 DPL.

### 5.9. Analysis of Cellular Distribution

Cells immuno-positive for Tuj-1, GFAP, Iba1, NG2 or MBP were quantified from respective fluorescent micrographs. Here, 5 regions per culture were selected using the DAPI-only channel on the fluorescent microscope; a minimum of 100 nuclei were assessed per condition. The percentages of each type were calculated by counting the proportion of cell marker-positive cells compared with total nuclei within the field.

### 5.10. Astrocyte Morphology and Analysis of Astrogliosis

Astrocyte morphologies were separated into 3 categories: finely processed astrocytes with small central soma (type 2 astrocyte), short-processed astrocytes with restricted processes and unprocessed astrocytes presenting as a spherical cell. The percent of each astrocyte morphology category was calculated for each condition.

To measure astrogliosis at the lesion site of the gels, the optical density of GFAP expression was evaluated. Firstly, fluorescent images were converted to 8-bit and inverted on ImageJ (v1.53) and the programme was calibrated for optical density. Next, using the freehand drawing tool, astrocytes at the lesion margins were traced and the overall optical density within the trace was calculated. A background optical density was also taken from an astrocyte-free area. The background OD was then subtracted from the OD of each astrocyte for that image. This was repeated for distal astrocytes over 400 µm from the lesion edge. The same exposure was used for imaging both lesional and distal areas of the gels. An average from each biological repeat was plotted for statistical analysis.

### 5.11. Analysis of Microglial Morphology

Microglial morphology within the 3D injury modelling system was characterised by a cell roundness index (CRI) [27]. This can quantitatively describe the effect of the biomaterial on the morphology of the resident microglia, to determine their possible reactive state. Iba1 immunolabeled micrographs were imported into ImageJ. The relevant scale was set globally, and the freehand drawing tool function selected. Each Iba1+ve cell within the analysed areas was traced around and measured. Recorded measurements from each traced cell provides cell perimeter and area values. These values were then plugged into the equation for CRI: CRI = 4π × Area/perimeter^2^; here the value of 1 denotes a complete circle, and towards 0 is ramified. It is important to note that since microglia were often distributed in patches throughout the gels, injuries did not always fall near or within a microglial patch. Though this rarely occurred, injuries without microglia nearby were not including within the quantification.

### 5.12. Statistical Analysis

All data are expressed as mean ± standard error of the mean (SEM). Data sets were analysed using Prism software (v5.0, GraphPad, USA). Note that *n* = number of constructs derived from independent litters of animals. A combination of unpaired two-tailed *t*-tests and one-way ANOVA statistical tests were employed and specified in the Results.

## Data Availability

The original contributions presented in the study are included in the article, further inquiries can be directed to the corresponding author.
